# Indents

**Published:** 1906-02-15

**Authors:** 


### INDENTS.

☞ **Brief Articles of Technical or Scientific Value will receive notice and comment. Contributions invited.**

**Sterilize Polishing and Grinding Stones.** Brush clean with soap and water with a plate brush, and then dip in a 5 percent solution of formalin in alcohol.

**Aluminum Solder.** For the flux use eighty parts stearic acid, ten parts cnloride of zinc, ten parts cnloride of tin. For tne solder use eignty parts of tin and twenty parts of zinc.—*Digest.*

**Wax for Naturai Bite Crown.** Melt a sheet of pink gutta-percha base plate into a melted sheet of pink base plate wax. Melt in an agate sauce pan and stir with a steel spatula until the gutta -percha is melted and thoroughly incorporated.—*Brief.*

**Home-made Gasoline Generator.** Take a Mason fruit jar and break out the porcelain on the inside of the lid; pass two suitable size brass tubes through this lid, one short the other extending to near the bottom of the jar. Solder them to the lid with soft solder. A foot bellows and blowpipe complete the outfit.—G. E. Frank, in *Brief.*

**Flask for Duplicating Plaster Models with Gelatine.** This mould consists of four pieces constructed of brass base plate, five and one-half inches in diameter with a groove one-sixteenth inch deep, extending around the plate one-fourth inch from the outer margins. Into this groove fits a ring of brass which is two inches high and one-sixteenth inch thick. The ring is made of two parts and held together by a metal band, assisted by the groove in the base plate. Thus it will be seen that the mould can readily be separated and gelatin, which is very tenacious, be easily removed after setting.—H. S. Keeler, in *Brief.*

**Antiseptic Liquid Soap.** Take, white soap, three pounds; glycerine, one-half pint; salol, two ounces; olive oil, one half pint; black soap, six ounces; water, three quarts.

Boil for three hours and cool for one day, then add three quarts of water, decant, cool and add one-half pint of alcohol. Let stand for two or three days and then filter. A few drops will clean and sterilize the hands.—Dr. H. L. Weber, in *Cosmos.*

**Antral Abscess. Diagnosis.** If an antral abscess is suspected let the patient blow his nose thoroughly upon the affected side until no pus is expelled. Then let him, with feet together, endeavor to touch his toes without bending the knees, for from one to two minutes. Extensive aching of the diseased antrum and eventually of the diseased teeth will be induced, and a stream of pus will result from renewed blowing of the nose.—H. Stolte, in January *Cosmos.*

**Making Gold Foil Cohesive.** Some time ago Mr. John Hood, of Boston, in response to an appeal for help in the difficulty directed me to follow the methods which the foil manufacturers themselves use in developing the full cohesiveness of gold. He kindly provided me with a large sheet of mica, about eight inches square, directed me to put it over a large clear bunsen flame, and upon it place the sheet of gold smooth and flat, letting it remain a minute or so. He said that however old the foil might be this would restore its cohesiveness. I annealed in this way a very few sheets at a time shortly before they are to be used, and I have been much gratified with the cohesiveness which has been developed in foil in which it had previously been lacking.—C. A. Brackett, in January *Brief.*

**Errors in Practice.** Inserting amalgam filling in proximal cavities without the matrix, resulting in packing the inter-proximal space and leaving the margins of the cavity rough and unprotected. Inserting gold fillings in incisors and cutting away freely the labial surface for access, but leaving thin lingual walls to fracture or leak. Failure to keep clean and sterile mouth mirrors, rubber dam holders, pliers, polishing stones and other facilities used on every patient.—S. A. Houchen, in *Brief*.

**Replacing a Porcelain Facing.** If for a bridge or crown cut off the pins of the broken facing and drill through the backing holes larger than the original platinum pins. Select a new porcelain facing and fit to place. Then cut off its pins to one-sixteenth of an inch of the facings and solder to them pure gold seamless tubes, long enough to reach through the backing and of size to fit the holes drilled. Countersink the holes on the lingual surface of the backing and set the facing with thin cement, smeared on the face of the backing and into the holes. Before the cement sets, with a pear-shaped burnisher in the engine, expand the lingual ends of the tubes to fit the countersunk holes, while holding the facing securely in place. This process rivets the tube pins and securely fastens the facing. The counter sunk ends may be filled and made smooth.—*Texas Journal.*

**A Prescription for Facial Neuralgia.** Twenty years ago I began experimenting to find a remedy, or a combination of remedies, that would cure facial neuralgia and at the same time one that would not exert a baneful influence upon the animal economy. In my experimental researches I have used over one hundred different single remedies, giving each a thorough test, with the result that many of them would benefit some cases, while in other cases the same remedy would have no effect on the complaint.

I found that compound remedies are more curative than single remedies. For one combination I have written over 300 prescriptions during the last ten years, and it has given perfect satisfaction to my patients and to myself, as it is a positive cure for nerveache of the fifth nerve, and will not disappoint you. The prescription is:

℞ Lloyd's specific aconite, twenty drops.
Lloyd's specific rhus tox, twenty drops.
Lloyd's specific gelsemium, one dram.
Fluid extract tongo, two drams.
Elixir aromatica, sufficient to make four ounces.

Of this give one dram every two hours until relieved: then for every four hours until cured. It will take from three to five doses to bring the system under the influence of the remedy, which will be manifest by a tingling sensation in the ends of the fingers. The above is the adult dose. The intelligence of the physician will modify the dose to the requirements of children.—*Chicago Medical Lines.*

**Gold Inlays.** Dr. A. H. Fleming describes in the Januaiy *Cosmos*, a method of making gold inlays. The cavity prepaiation is the same as usual. The matrix is carefully made of pure gold, three one-thousandths of an inch in thickness, after it is carefully adapted it is filled to contour with warmed modeling compound, and when cold and hard the matrix is removed and invested in any good investment material to the cavity margin of the matrix. The compound is then warmed and removed, and a mixture of nine parts of plaster of paris and one part of fine soapstone is then worked into a thick mass with alcohol and placed into the matrix where the modeling compound was and to the same contour. A portion of this mixture after it has set is carved away to the thickness wanted for the gold surface of the inlay, and the original contour is again restored with moss fibre gold. A little cream borax is then placed on this gold, and the invested inlay is placed over a low bunsen flame until it is thoroughly dried and heated to a temperature where solder can be flowed with a blowpipe over the entire surface of the moss fibre gold filling. The solder is absorbed and a smooth solid surface results. When sufficient solder is applied, withdraw the bunsen flame and move a brush flame of the blowpipe back and forth over the gold surface until a perfect glaze is obtained. Remove the inlay from investment and polish to the margins. Open through the matrix and remove the plastic core and it is ready to set. The cavity left by removing the core should be filled with the cement used in setting.

**What Prophylaxis Means.**

That the administration of prophylactic treat, according to the methods now coming into recognition, requires arduous service, no one who has sought faithfully to employ it will deny. Consequently, that often re-curring question of compensation is a great barrier to its general introduction; but the fact that we can consistently promise our patients that through its partial prevention of caries it is not only not expensive, but is splendidly economic, will, in time, remove this barrier. The compensation which is the goal of the truly professional man is not entirely material, but must be, in part, altruistic. Rub, young man, rub; rub your way to your patient's hearts and influence; rub and reach their pockets, too. Teach your clients that sacrifices are due to Hygeia's altar as well as at other shrines, and, too, that sacrifices continually made become oblations. And fail not to teach the same to yourself.

Rub and educate and strengthen your hand for the battle that's before you. And, rubbing, behold, in the swift course of time, the indescribable beauty of a hygienic human mouth —glittering pearl and shining ivory and ruddy coral, in nature's matchless profusion of curved lines—but we cannot paint the lily nor can we gild refined gold. "Breathes there a man with soul so dead!" Rub, my brother, rub; "what cannot art and industry perform when science plans the progress of their toil." "Learn to labor and to wait." One of the most satisfactory services the author has ever done in his professional career was the separation of a molar from a bicuspid which it held in the hideous embrace of an enormous cavity which contained a fourth of the bicuspid's crown, and the insertion and polishing of an amalgam filling to restore the normal relations of the two organs and a hygenic condition of the contiguous parts, a week of time and seven visits being required to accomplish the separation. —J. H. Crossland, in *Dental Brief.*

**Stanno-Percha.** All the preparations heretofore in the market possess the disadvantages of undergoing considerable change in form shortly after insertion in the cavity. The mass bulges out and, exposing the cavity margins, renders possible the recurrence of the carious processes. Furthermore the insertion of a gutta-percha filling is by no means an easy operation, either because it is as a rule necessary to hold the first pieces in place by means of one instrument while with another the mass is adapted to the walls of the cavity, or else because particles of the filling material adhere tenaciously to the instrument. To obviate the above enumerated disadvantages, Dr. Scheurer recommends the tin gutta-percha combination suggested.

The form of tin which is incorporated into the gutta-percha is the so-called sponge tin suggested by Dr. Scheuer about two years ago. It is prepared by dissolving one hundred grams of chemically pure stannic chlorid in one liter of water. The precipitation of the tin is brought about by suspending in the solution a plate of chemically pure zinc. The precipitate must be washed repeatedly and also boiled until the water shows no cloudiness whatever, the purpose of this thorough washing being to remove all particles of zinc chlorid. The washing should be continued until the litmus-paper test shows that all traces of acid have been removed. Stanno-percha is prepared by mixing in a mortar in a sand bath equal parts of pink gutta-percha and sponge tin. The two ingredients should be thoroughly rubbed until the whole mass assumes a grayish color. One-half of the mass is then removed from the mortar and to the remaining half is again added an equal amount of gutta-percha. The mass at first removed from the mortar is soft and is used to line the cavity, while the material which remained in the mortar and which was mixed with an additional amount of tin is used to complete the filling. A filling material thus prepared possesses several advantageous features, inasmuch as it makes a dense filling, is not affected by friction, does not expand under the influence of its humid surroundings, becomes soft at a relatively low temperature, and remains so longer than the unmixed material, thus obviating the necessity of heating the instruments repeatedly. It posseses also over the older preparations the valuable feature of not adhering to the instrument when heated previously to being inserted in the cavity. Stanno-percha can be used in combination with gold or amalgam alloys. The deeper portions of the cavity are filled with stanno-percha, which acts as a protective lining to the pulp, and the filling is completed with either gold or amalgam, which materials adapt themselves perfectly to stanno-percha, especially gold when hot through annealing.—*Cosmos.*

**Causes of Antral Diseases.** It used to be the general opinion that all cases of antral abscess were caused by diseased teeth. Strubel of Dresden, has, in a very exhaustive paper, studied this question, and collected the opinions of all the writers in the world's literature on this subject: He comes to this conclusion: 64:6 percent were undoubtedly of nasal origin; 29 percent of possible dental origin; 3.2 percent traumatic; 3.2 percent luetic.

With regard to the dental origin of the antral abscess, we can conclude—if the nose be absolutely sound, if influenza or the like can be ruled out, and where there is nothing to be considered except the diseased tooth or clear history of it (chronic alveolar abscess, or cysts, or alveolar pyorrhea) —it is more than probable, from the clinical standpoint, that the tooth was the cause.

The most common cause for antral infection, so far as the teeth are concerned, is that resulting from chronic alveolar abscess, chronic alveolar periostitis, cysts of the roots with purulent contents, and, more seldom, pyorrhea alveolaris. When the diseased root has done its injurious work the alveolar abscess or the periostitis may heal, and yet the antral abscess persist as an independent disease, increasing gradually in extent, the cavity being filled out with granulation tissue and polypi. The fetid pus issuing continually through the antral foramen into the infundibulum of the middle meatus produces edematous swelling of the mucous membrane of the infundibulum and surrounding parts, and later on polypus degeneration and proliferation. In that way the already narrow nose passage become more obstructed, and the purulent discharge is gradually forced into all the other sinues when the patient tries to free the nose-passage from the molesting discharge by blowing.

The value of decayed teeth as an etiological factor is often over estimated; for instance by Tilley, who figured that 90 percent of his antral abscess cases were caused by decayed teeth, inasmuch as 90 percent of his patients chanced to have decayed teeth. But as we know that a simple decayed tooth with living pulp, without affection to the alveolar membrane, without abscess or periostitis, can never produce an antral abscess. The mere coincidence of the presence of decayed teeth in many antral cases cannot be used as proof of the dental origin of the antral abscess; for when we ought to meet with antral abscess, for instance, in England or Germany or in nearly all continental countries, as a quite common disease, while, as it is, the number of antral abscesses in those countries, where nearly 80 percent of all adults have some decayed teeth, is in average not much larger than in the United States, where are found, owing to the excellent attention to and care of the teeth on the part of the dental profession, the healthiest and soundest teeth in the world.

With regard to traumatism as an etiological factor, I cannot deny that careless operation by a dentist sometimes causes infection of the sinus, as from forcing the root of tooth into the sinus through fracture of the wall, in an unfortunate effort to extract, or in driving artificial crowns or bridges upon the teeth or roots, or drilling through the root into the sinus.—H. Stolte, in January *Cosmos*.

